# N-terminal modifications of cellular proteins: The enzymes involved, their substrate specificities and biological effects

**DOI:** 10.1002/pmic.201400619

**Published:** 2015-06-16

**Authors:** Sylvia Varland, Camilla Osberg, Thomas Arnesen

**Affiliations:** 1 Department of Molecular Biology, University of BergenBergen, Norway; 2 Department of Surgery, Haukeland University HospitalBergen, Norway

**Keywords:** α-amino group, Acetylation, Cell biology, N-terminal, Protein modification, Substrate specificity

## Abstract

The vast majority of eukaryotic proteins are N-terminally modified by one or more processing enzymes. Enzymes acting on the very first amino acid of a polypeptide include different peptidases, transferases, and ligases. Methionine aminopeptidases excise the initiator methionine leaving the nascent polypeptide with a newly exposed amino acid that may be further modified. N-terminal acetyl-, methyl-, myristoyl-, and palmitoyltransferases may attach an acetyl, methyl, myristoyl, or palmitoyl group, respectively, to the α-amino group of the target protein N-terminus. With the action of ubiquitin ligases, one or several ubiquitin molecules are transferred, and hence, constitute the N-terminal modification. Modifications at protein N-termini represent an important contribution to proteomic diversity and complexity, and are essential for protein regulation and cellular signaling. Consequently, dysregulation of the N-terminal modifying enzymes is implicated in human diseases. We here review the different protein N-terminal modifications occurring co- or post-translationally with emphasis on the responsible enzymes and their substrate specificities.

## 1 Introduction

**Figure 1:**
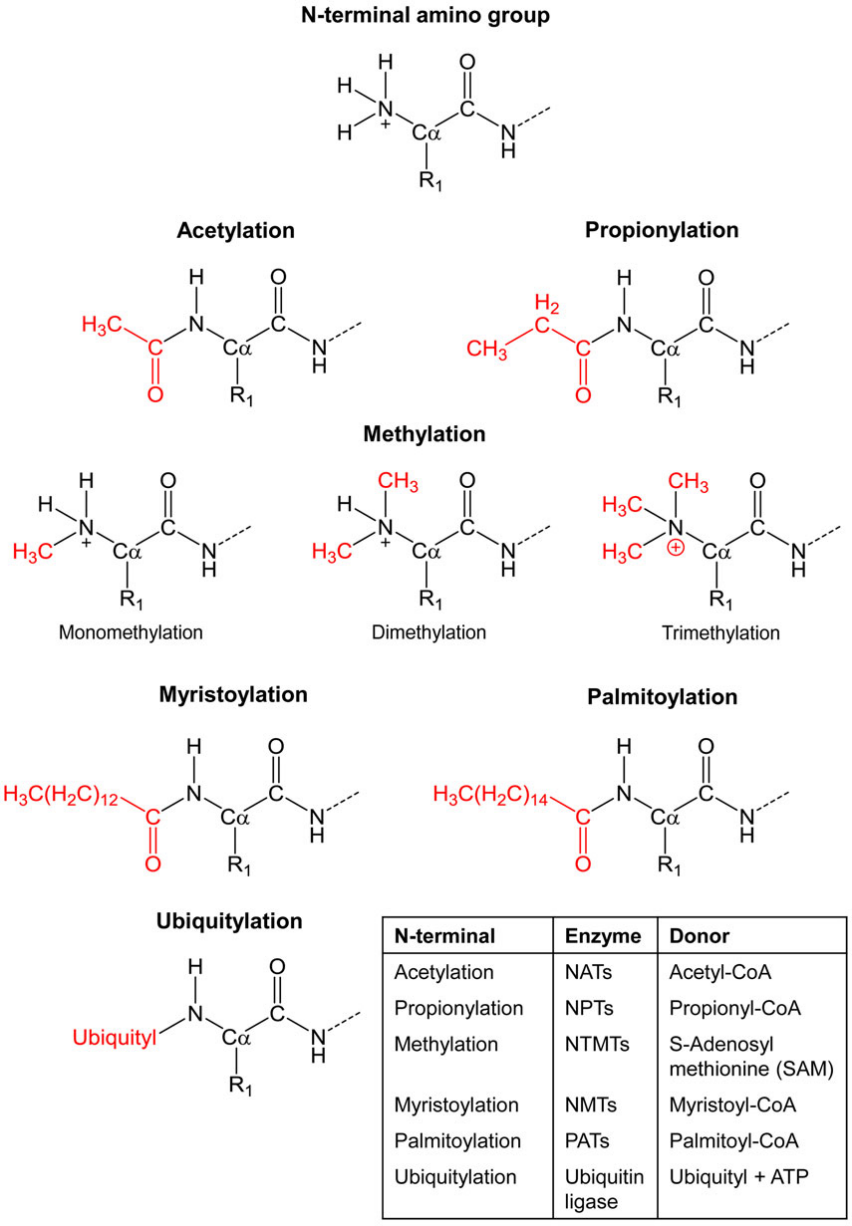
Structural formulae of major N-terminal protein modifications. The N-terminal α-amino group is (usually) positively charged at neutral pH. The chemical character of protein N-termini can be modified by, for example: acetylation, propionylation, methylation, myristoylation, palmitoylation or ubiquitylation (attachment shown in red). The responsible enzymes and their respective donor molecules are listed. NATs, N-terminal acetyltransferases; NPTs, N-terminal propionyltransferases; NTMTs, N-terminal methyltransferases; NMTs, N-terminal myristoyltransferases; PATs, Palmitoylacyltransferases; CoA, Coenzyme A; ⊕ denotes permanent positive charge.

From the moment a eukaryotic nascent polypeptide emerges from the ribosome, a machinery of different enzymes is in place to modify its N-terminal (Nt) amino acid residue. These modifications (Fig. 1) have evolved to substantially increase the cellular protein repertoire. Despite the abundance of Nt-modifications, the specific functions of N-terminally modifying enzymes remain incompletely understood. Initiator methionine excision by methionine aminopeptidases (MetAPs) is very common and essential, but not comprehended in terms of its functional implications [1]. Another highly abundant co-translational modification is Nt-acetylation catalyzed by N-terminal acetyltransferases (NATs) [2]. The NATs also carry out Nt-propionylation, a much rarer and less understood modification [3]. Protein fatty acylation of the N-terminus normally involves Nt-myristoylation catalyzed by N-terminal myristoyltransferases (NMTs) [4]. Nt-palmitoylation is rarer and is carried out by distinct enzymes, the N-terminal palmitoylacyltransferases (PATs) [5]. The abovementioned modifications mostly occur co-translationally, but the N-terminus can also be post-translationally modified. Nt-methylation is a common type of post-translational modification catalyzed by N-terminal methyltransferases (NTMTs) [6,7]. Additionally, Nt-ubiquitylation has emerged as a new scarce member of the Nt-modification family [8]. Together, the various Nt-modifications have profound functional effects. The responsible enzymes act in more or less sequence specific manners in order to establish specific functions to particular substrate proteins. We will here review the major co- and post-translational protein Nt-modifications including their biological impact. We will further address the current knowledge of the responsible enzymes and their substrate sequence requirements.

## 2 N-terminal methionine cleavage

When a nascent polypeptide emerges from the ribosomal exit tunnel it is introduced to a set of different proteins. Amongst them is MetAPs methionine aminopeptidases (MetAPs) [9], destined to co-translationally remove the initiator methionine (iMet) in the case of a favourable second amino acid. Complete cleavage of the iMet is achievable when Ala, Cys, Gly, Pro, Ser, Thr or Val sequesters the second position (Table 1). These amino acids all have an uncharged side chain with a radius of gyration of <1.29 Å as defined by Levitt [10]. In contrast, there is a full iMet-retention when the second residue belongs to any of the other naturally occurring amino acids. These features make up the criteria for iMet removal on a nascent polypeptide [1,10–14]. However, in some cases the amino acid residue in the third position can influence the activity of MetAPs. For example, the iMet of the β-chain of Hemoglobin Long Island [15,16] and Hemoglobin Marseille [17] is retained following His to Pro substitution at position three, possibly due to steric hindrance. Some actins constitute an exception to the general iMet excision pathway. For instance, mammalian cytoplasmic γ-actin (Met-Glu-Glu-) has a large amino acid residue in the second position and the iMet is not processed by the co-translationally acting MetAPs. Instead, this actin is post-translationally processed by an unidentified aminopeptidase [18].

**Table 1:** Protein N-terminal modifications specified by the responsible enzymes and their substrate specificity

Nt-modification	Enzyme	Proteinb)	Alternative name	UniProt	Substrate specificityc)
iMet excision	MetAP	MetAP1 MetAP2	MAP1A, Peptidase M 1MAP2, MNPEP, P67EIF2	P53582 P50579	Met-Ala-, Met-Cys-, Met-Gly-, Met-Pro-, Met-Ser-, Met-Thr-, Met-Val-
		*ND*			Met-Asp-, Met-Glu-
Acetylation Propionylationa)	NatA	Naa10^cat^	ARD1, ARD1A, TE2	P41227	Ala-, Cys-, Gly-, Ser-, Thr-, Val-, Aspd)-, Glud)-
		Naa15^aux^	NAT1, GA19, NARG1, NATH, TBN	Q9BXJ9	
	NatB	Naa20^cat^	NAT3, NAT5	P61599	Met-Asn-, Met-Asp-, Met-Gln-, Met-Glu-
		Naa25^aux^	MDM20, NAP1	Q14CX7	
	NatC	Naa30^cat^	MAK3, NAT12	Q1473	Met-Ile-, Met-Leu-, Met-Phe-, Met-Trp-
		Naa35^aux^	MAK10, EGAP,	Q5VZE5	
		Naa38^aux^	MAK31, LSMD1, PFAAP2	Q9BRA0	
	NatD	Naa40^cat^	NAT4, NAT11	Q86UY6	Ser-Gly-Gly-, Ser-Gly-Arg-
	NatE	Naa50^cat^	MAK3, NAT13, SAN	Q9GZZ1	Met-Ala-, Met-Leu-, Met-Lys-, Met-Phe-, Met-Ser-, Met-Thr-, Met-Tyr-, Met-Val-
	NatF	Naa60^cat^	NAT15, HAT4	Q9H7X0	Met-Ala-, Met-Gln-, Met-Gly-, Met-Ile-, Met-Leu-, Met-Lys-, Met-Met-, Met-Ser-, Met-Thr-, Met-Tyr-, Met-Val-
Methylation	NTMT	NTMT1	METTL11A, NRMT1, NRMT1A	Q9BV86	Ala/Pro/Ser-Pro-Lys-
		Tae1 (*S.c*)	NTM1	P38340	
		NTMT2	METTL11B, NRMT2, NTM1B	Q5VVY1	
Myristoylation	NMT	NMT1	NMT	P30419	Gly-
		NMT2		O60551	
Palmitoylation	PAT	Hhat	MART2, SKI1, Skn	Q5VTY9	Cys-
		Rasp (*D.m*)	cmn, sit, ski	Q9VZU2	
		*ND*			Gly-
Ubiquitylation	Ubiquitin	Ube2w	UBC16, UBC-16,	Q96B02	Unstructured N-terminal backbone
	ligase	HUWE1	ARF-BP1, HectH9, LASU1, Mule, UREB1, URE-B1	Q7Z6Z7	*ND*

a) N-terminal propionylation is catalyzed by the same enzymes as N-terminal acetylation (NATs), and the substrate specificity is presumably shared.

b) All proteins listed are human except where the species is indicated.

c) The indicated amino acid sequences do not guarantee N-terminal modification.

d) Naa10 substrates.

*ND*, not determined; Aux, auxiliary subunit; Cat, catalytic subunit; *S.c*, *Saccharomyces cerevisiae*; *D.m*, *Drosophila melanogaster*.

The event of iMet removal is conserved throughout evolution [19] and is predicted to occur when the nascent polypeptide has reached a length of 20–40 amino acids [20–22]. It is estimated that more than 50% of all proteins are subjected to iMet removal [23]. Arfin et al. proposed a model for the subfamilies of MetAPs based on the catalytic cobalt-binding domain of the enzymes [24]. The updated version of this model, based on additional sequence features like N-terminal extension, linker region and zinc finger domains, displays two groups of MetAPs, namely MetAP1 and MetAP2. The former is divided into four subgroups, Type 1a-d. Type 1a and 1c are found in prokaryotes, while Type 1b, 1d and Type 2 MetAPs are present in eukaryotes [19,24–32]. Predictions on ribosomal binding of MetAPs suggest that one or several exposed PXXP motifs of the peptidases are involved in protein-protein interactions [25,26,33].

Structural studies of human MetAP1 and MetAP2 revealed a potential difference in the substrate specificity of the respective catalytic sites due to more steric restrictions in MetAP1 [25]. This is supported by the finding that MetAP2, and not MetAP1, is inhibited by the anti-angiogenic agent fumagillin [32]. Growth studies in yeast have shown that the iMet processing provided by MetAP1 and MetAP2 is essential since a double deletion is lethal for yeast while the single deletions are viable [30]. This strongly suggests that both enzymes, at least in part, act on the same substrates in vivo. Significant overlap in substrate specificity was also found for human MetAP1 and MetAP2, although a significant preference of MetAP2 for Met-Val- and Met-Thr- N-termini was observed [34].

## 3 N-terminal acetylation

One of the most abundant protein modifications occurring in eukaryotes is N-terminal acetylation (Nt-acetylation), where an acetyl moiety is transferred from acetyl-CoA to the α-amino group of a nascent polypeptide. The Nt-acetylome is estimated to include 80–90% of soluble human proteins and 50–70% of yeast proteins [35,36]. N-terminal acetylation of a specific protein can either be complete or partial, and in the latter case the protein exists in both acetylated and non-acetylated forms.

Traditionally, Nt-acetylation has been regarded as a co-translational process taking place on a nascent polypeptide when approximately 25–50 amino acids residues emerge from the ribosomal exit tunnel [37,38]. In addition, there are examples of post-translational Nt-acetylation of proteins [39,40]. The acetylation process neutralizes the positive charge normally associated with the free α-amino group, and thereby efficiently blocks the α-amino group for further ionization and other modifications (Fig. 1).

Nt-acetylation is catalyzed by a set of enzymes, the N-terminal acetyltransferases (NATs). The NATs are members of the GNAT protein superfamily [41], all containing the consensus acetyl-CoA binding sequence (Q/R)XXGXX(G/A) [42]. The enzymatic machinery is conserved from lower to higher eukaryotes suggesting a comparable system for Nt-acetylation [43]. The various NATs display different substrate specificities (Table 1), largely attributed to the identity of the first two amino acids in the polypeptide sequence [44]. However, residues beyond position seven may have an influence [35].

The majority of Nt-acetylation is catalyzed by NatA, NatB and NatC. Here, the NAT-function is dependent upon complex formation between a unique catalytic subunit and one or two auxiliary subunits. The auxiliary subunits have various functions including ribosomal anchoring [45,46]. NatA, the major NAT-complex, is composed of the catalytic subunit Naa10 and the regulatory subunit Naa15 [47–49]. The NatA complex acetylates N-termini starting with Ala, Cys, Gly, Ser, Thr or Val following iMet removal [35,44]. In the absence of Naa15 the substrate specificity profile of Naa10 changes towards acidic N-termini [49]. The NatB complex, which is composed of the catalytic Naa20 and auxiliary Naa25 subunits [50,51], acetylates Met-Asn-, Met-Asp-, Met-Gln- and Met-Glu-starting N-termini [18,44,52]. NatC, on the other hand, is composed of the three subunits Naa30, Naa35 and Naa38. Naa30 is responsible for the acetylation reaction, but all subunits appear to be required for NatC-activity [53]. The activity of NatC is directed towards the iMet when followed by a hydrophobic second residue, that is Ile, Leu, Phe, or Trp [18,44,54,55].

Three additional NATs (NatD, NatE, and NatF) have been identified thus expanding the NAT-family substrate repertoire. The evolutionarily conserved acetyltransferase NatD displays a limited substrate profile by N-terminally acetylating only histones H2A (Ser-Gly-Arg-) and H4 (Ser-Gly-Gly-) [56,57]. However, recent data suggest that NatD may share some Ser-Gly- starting substrates with NatA [58]. NatE was first reported when yeast Naa50 was found to be physically associated with the NatA complex, but no impact on NatA-activity was observed [45]. In vitro studies revealed that human Naa50 is able to perform Nt-acetylation of a Met-Leu- substrate, and the activity was termed NatE [59]. The substrate specificity profile of NatE was later expanded to include Met-starting N-termini: Met-Ala-, Met-Lys-, Met-Met-, Met-Phe-, Met-Ser-, Met-Thr-, Met-Tyr-, and Met-Val- [49]. The evolutionary shift in the degree of Nt-acetylation between yeast and human could partly be explained by the presence of NatF (Naa60) in human (and other multicellular eukaryotes). NatF acetylates Met-Lys- N-termini which are rarely acetylated in yeast, in addition to Met-Ala-, Met-Gln-, Met-Gly-, Met-Ile-, Met-Leu-, Met-Met-, Met-Ser-, Met-Thr-, Met-Tyr- and Met-Val-. NatC, NatE and NatF thus have partially overlapping substrate specificities [36]. Interestingly, Aksnes and colleagues recently reported that NatF is localized to the Golgi, where it specifically Nt-acetylates transmembrane proteins, most likely in a post-translational mode [182]. This study also uncovered that Nt-acetylation is highly abundant among human transmembrane proteins.

Worth mentioning, the Nt-acetylation signatures are not absolute. The specificity can be affected by downstream residues and other determinants, such as secondary structures [18]. This could also explain the existence of both complete and partial Nt-acetylation. Goetze et al. have shown that Nt-acetylation is prevented when the nascent protein has a Pro in the first or second position. Pro as a definite determinant preventing Nt-acetylation is referred to as the (X)PX rule [60]. No N-terminal deacetylase has been identified and thus Nt-acetylation is considered irreversible.

The crystal structures of several NATs have been solved [61–65], including *S. pombe* NatA [63] and human Naa50/NatE [62]. Structural variability between the two catalytic subunits of NatA and NatE contributes to the substrate specificity. Binding of the auxiliary subunit may introduce conformational changes in the active site of the catalytic subunit and promote sequence-specific Nt-acetylation as seen in NatA [63]. Recent evidence suggests that Nt-acetylation takes place through an ordered ternary-complex (Bi-Bi) mechanism [62–64,66]. Here, binding of acetyl-CoA induces rearrangements in the NAT-enzyme, which subsequently increases the affinity for the peptide substrate allowing the reaction to occur.

## 4 N-terminal propionylation

The N-terminal protein modification family was recently expanded to include a new member, namely N-terminal propionylation (Nt-propionylation) (Fig. 1). Propionylated N-termini were first discovered in human cells [67,68] and later shown to occur in yeast, demonstrating that this modification is evolutionarily conserved [3].

Studies by Foyn and colleagues show that NATs may also function as N-terminal propionyltransferases (NPTs), both in vivo and in vitro. By challenging purified human Naa10, Naa50, and NatA with propionyl-CoA they demonstrated that all enzymes could indeed perform Nt-propionylation on substrate peptides in vitro. The NPT-activity of purified Naa10 and Naa50 were far less efficient than their acetylation activities using acetyl-CoA. Intriguingly, the NatA complex performed Nt-acetylation and Nt-propionylation with similar rates, particularly for peptides substrate starting with Ser. Furthermore, Nt-proteomics revealed that both yeast and human NatA complexes could perform Nt-propionylation in vivo [3].

Since propionylation of protein N-termini was only recently discovered, virtually nothing is known about its functional importance. The cellular level of acetyl-CoA is 2-20-fold higher compared to propionyl-CoA [69,70]. This could explain the low detection rate of Nt-propionylated compared to Nt-acetylated substrates. In addition, it is uncertain whether Nt-propionylation occurs on specific substrates and conveys a signal different from Nt-acetylation. The propionyl group contains an additional methyl moiety as compared to acetyl and this might result in additional bulkiness and hydrophobicity. Hitherto 18 proteins have been identified as being Nt-propionylated, four of which are processed mitochondrial proteins. This indicates a post-translational as well as a co-translational mode for this modification [3,67,68].

## 5 N-terminal myristoylation and palmitoylation

N-terminal myristoylation (Nt-myristoylation) refers to the irreversible transfer of myristoyl (14-carbon saturated fatty acid) from myristoyl-CoA to the N-terminal Gly of the target protein (Fig. 1). N-terminal myristoyltransferase (NMT) catalyzes the chemical reaction [4], following an ordered Bi-Bi reaction mechanism [71–74]. Initially, this protein modification was found to exist as a co-translational event, supported by the isolation of myristoyl-labelled nascent polypeptide chains associated with tRNA and the identification of NMT in the ribosomal subcellular fraction [75–78]. However, Nt-myristoylation has been found to occur as a post-translational event as well, mainly in apoptotic cells [79–81].

Prior to Nt-myristoylation, the iMet needs to be removed by MetAP, thus exposing the consensus sequence recognized by NMT. The consensus sequence has been revealed in the context of several in vivo and in vitro studies [4,82]. Gly in the first position is an absolute requirement. In the second position a charged residue is favored, whereas aromatic residues and Pro are prohibited. There are no special requirements for the third position. Ala, Asn, Cys, Gly or Ser is allowed in the fourth position while in position five Cys, Ser, or Thr is preferred and Pro is prohibited.

Two isozymes of NMT, NMT1 and NMT2, have been isolated and characterized [76,83,84]. The evolutionary conservation of both structure and substrate specificity (Table 1) [82,84–90]. Human NMT1 and NMT2 possess approximately 77% amino acid sequence identity. The majority of divergence between the two NMTs is seen in the N-terminal domain, which is important for ribosomal binding [84,91]. Both unique and overlapping substrate specificities are observed between NMT1 and NMT2 within a given species [84,90,92–94].

It is estimated that about 0.5% of cellular proteins are Nt-myristoylated [95–99]. Different Nt-myristoylation prediction tools exist, e.g. MYR Predictor [100] and Myristoylator [101]. Caution should be exercised because false-positive and false-negative predictions might occur [102], thus demonstrating the necessity of both in vivo and in vitro studies for complete data [4]. Thinon et al. recently established a global profile of the Nt-myristoylome in both normal and apoptotic cells [103].

Unlike the immense amount of data on Nt-myristoylation, far less is known about Nt-palmitoylation. Normally, the palmitoyl group (16-carbon saturated fatty acid) is attached to an internal Cys residue [104], but a few instances of palmitoylated N-termini have been uncovered. A study by Klauss and Krause shows co-translational Nt-palmitoylation of Gα_S_, the α-subunit of the heterotrimeric G protein responsible for activation of adenylyl cyclase. Here, a palmitoyl group from palmitoyl-CoA is attached to the α-amino group of the N-terminal Gly residue (Fig. 1) [105,106].

Interestingly, the secreted vertebrate signaling proteins Hedgehog (Hh) and Sonic Hedgehog (Shh) have been found Nt-palmitoylated at Cys, following cleavage of the N-terminal signal sequence [5,107]. Hhat, an N-terminal palmitoylacyltransferase (PAT) is suggested to palmitoylate Shh [108–111], and this modification constitutes an important regulatory feature for the strength of Shh signaling [112]. Hhat belongs to the family of multipass transmembrane proteins termed MBOAT (membrane-bound O-acyltransferase) [113] and acylates Shh during its passage through the secretory pathway [108]. Rasp, another member of the MBOAT family, is responsible for Nt-palmitoylation of the Hh and Spitz proteins in *D. melanogaster* [109,114,115].

## 6 N-terminal methylation

N-terminal methylation (Nt-methylation) is a process by which a methyl group is transferred from S-adenosyl methionine (SAM) to the exposed N-terminal α-amino group following iMet cleavage. The existence of Nt-methylation has been known for decades [6,116–118], however its structural and functional importance has only recently emerged [119–124].

The chemical consequence of Nt-methylation depends on the degree of residue methylation. Monomethylation will probably have a minor effect on the basicity of the α-amino group, by increasing the pK_a_ slightly and cause some steric hindrance that may reduce its reactivity. In contrast, trimethylation (or dimethylation in the case of Pro) will have a profound effect by generating a permanent positive charge on the N-terminal amino group (Fig. 1). Consequently, the nucleophilicity normally associated with the α-amino nitrogen is abolished [6]. Nt-methylation is assumed irreversible as no N-terminal demethylase has been identified [7].

Recently, two independent studies finally identified the first N-terminal methyltransferases (NTMTs). The orthologues yeast Tae1 and human METTL11A were reported to respectively catalyze the stoichiometric Nt-methylation of the ribosomal proteins Rpl12a/b and Rps25a/b [125] and RCC1, RB and SETα [126]. To reflect their unique role in Nt-methylation the enzymes were renamed NTMT1. NTMT1, which is a member of the seven-beta-strand class I methyltransferase family, is conserved across eukaryotes and have one close human homologue. A study by Petkowski et al. confirmed that NTMT2 displays N-terminal methyltransferase activity as well [127].

The substrate consensus sequence for the eukaryotic NTMTs was initially thought to be X-Pro-Lys-, where X can be Ala, Pro or Ser (Table 1) [6]. Further in vitro studies have shown that NTMT1 is somewhat promiscuous concerning the identity of the first amino acid. A recombinant NTMT1 is able to methylate RCC1 peptides as long as the first position is not occupied by the acidic residues Asp or Glu, Trp, or the hydrophobic residues Ile or Leu [126]. An expanded peptide library methylation assay furthermore showed that the presence of a Pro in the second position is not an absolute requirement [128]. Efficient Nt-methylation requires Lys in the third position, but can in rare cases be replaced by Arg [123,126,128]. Taken together this implies that NTMT1 may have a broader specificity than previously acknowledged, and this could also be the case for NTMT2. Interestingly, it was recently shown that CENP-A [123] and CENP-B [121] with the Gly-Pro-starting N-termini are Nt-methylated in vivo.

Given both sequence and structural similarity [127], it is not unreasonable to believe that NTMT1 and NTMT2 have similar localization patterns and catalytic activities. In fact, both enzymes are expressed at low levels and localizes predominantly to the nucleus [127]. In contrast, the enzymes display different methylation mechanisms. NTMT1 is a distributive trimethylase, which can mono-, di-, and trimethylate its substrates whereas NTMT2 is primarily an Nt-monomethylase. A synergistic NTMT mechanism has been proposed where NTMT2 primes substrates for subsequent di- and trimethylation by NTMT1. Hence, NTMT2 would confer aid to NTMT1 when the substrate burden is too high [127].

## 7 N-terminal ubiquitylation

The addition of ubiquityl to a substrate protein proceeds through a three-step process that is achieved by the combined activity of ubiquitin activating (E1), conjugating (E2), and ligating (E3) enzymes. Most commonly, ubiquityl is conjugated to the ε-amino group of an internal Lys residue. N-terminal ubiquitylation (Nt-ubiquitylation) refers to the addition of an ubiquityl moiety to the free α-amino group of the first residue of a protein (Fig. 1). In both cases, ubiquityl may serve as a target for polyubiquitylation, which is a well-known degradation signal recognized by the proteasome [129,130].

An N-terminal residue was initially found by Breitschopf et al. to act as a novel site for ubiquitylation when Lys replacement in the protein MyoD did not significantly affect its susceptibility for either in vitro or in vivo ubiquitylation or degradation [131]. The first direct evidence of Nt-ubiquitylation came when MS analysis revealed that ubiquityl was indeed fused to the N-terminal α-amino group of HPV-58 oncoprotein E7 [132]. Previously, there had been strong indications that a handful of proteins underwent degradation mediated by Nt-ubiquitylation [133–139]. However, in these cases the stability is presumably modulated through interplay between ubiquitylation at the N-terminus and on internal Lys residues. Seeing that HPV-58 E7 is a naturally occurring lysine-less protein its degradation is more likely to be completely dependent upon Nt-ubiquitylation.

The known substrates for Nt-ubiquitylation do not share any homology in their N-terminal region [8]. Thus, it is not unreasonable to consider that Nt-ubiquitylation is facilitated by several enzymes that provide different substrate specificity, subcellular localization and modes of regulation. Hitherto, E2 Ube2w [140,141] and E3 HUWE1 [142] are the only enzymes with a reported ability to ubiquitylate the N-terminus of substrates. HUWE1 was shown to ubiquitylate the N-terminus of lysine-less MyoD, but, interestingly, favors an internal Lys in wild-type MyoD and leaves the N-terminus unubiquitylated [142]. Ube2w, on the other hand, is able to successfully ubiquitylate the N-terminus of a lysine-less version of Ataxin-3 and Tau [140]. When comparing the active site of Ube2w to that of classical E2s there are some distinctive differences. Together, these features make the novel active site of Ube2w more suitable to accommodate a neutral α-amino group rather than a positively charged Lys side chain [140,141]. Interestingly, Vittal et al. recently reported that Ube2w recognizes the peptide backbone of unstructured N-termini, and that the presence of Pro in position two to four has an inhibitory effect on Ube2w-activity [143].

Nt-ubiquitylation is not to be confused with the N-end rule, which relates the in vivo half-life of a protein to the identity of its N-terminal residue. Specific E3 ubiquitin ligases, called N-recognins, target protein substrates through their destabilizing N-terminal residues for polyubiquitylation and proteasomal degradation [144,145]. In eukaryotes, these N-terminal degradation signals, called N-degrons, comprise a destabilizing N-terminal residue as well as an internal lysine(s) residue within an unstructured (flexible) segment of the protein's polypeptide chain [146]. Primary destabilizing residues are positively charged (basic) and bulky hydrophobic N-terminal residues that are directly recognized by N-recognins [147,148]. A recent study reported that an unacetylated N-terminal Met could also act as a primary destabilizing residue if this Met is followed by a hydrophobic residue [149]. The secondary and tertiary destabilizing residues Asn, Asp, Cys, Gln, and Glu require preliminary enzymatic modifications, including Nt-deamidation and Nt-arginylation, before the recognition by N-recognins of the N-end rule pathway [147,148]. Yet another mechanistically distinct branch of the N-end rule pathway was identified in 2010 [150]. In this branch, termed the Ac/N-end rule pathway (the previously known branch was termed the Arg/N-end rule pathway), destabilizing N-terminal residues (those that can be Nt-acetylated by NATs) are recognized by distinct ubiquitin ligases (Doa10 and Not4) termed Ac/N-recognins [150,151]. The N-end rule pathway continues to be deciphered and as novel features are revealed, the framework of this proteolytic system keeps expanding (recommended in-depth reviews for further reading [145,147,148,152]).

## 8 Biological functions of N-terminal modifications

While the presence and abundance of Nt-modifications have been thoroughly demonstrated during the last decades, the functional roles of these modifications are now beginning to emerge. Very recent studies demonstrated that iMet cleavage and Nt-acetylation might be important players in the expanded N-end rule pathway, linking the identity of the protein N-terminus to its in vivo stability [149–151,153]. Nt-acetylation may further be important for targeting specific proteins to intracellular membranes like the Golgi-membrane or the inner nuclear membrane [154–156]. In a global survey, N-termini of cytosolic proteins were found to be more prone to be Nt-acetylated as compared to secreted proteins [157]. Interestingly, mutating the N-termini of specific proteins with signal sequences to become Nt-acetylated inhibited their post-translational translocation to the endoplasmic reticulum. This suggested that Nt-acetylation prevents this type of subcellular targeting. In some cases, Nt-acetylation is crucial for proper protein complex formation [158,159]. A very recent investigation revealed that deficiency in NatA-mediated Nt-acetylation most likely causes misfolding of a variety of NatA substrates thus suggesting Nt-acetylation to be a general factor mediating protein folding [160–162]. The (patho)physiological importance of Nt-acetylation only recently emerged, as the Ogden syndrome was found to be caused by a NAT mutation [58,163,164]. Clinical features of the Ogden syndrome include postnatal growth failure, developmental delays and death during infancy [164]. The genetic cause of the Ogden syndrome, a Ser37Pro mutation in *NAA10*, results in impaired enzymatic activity and NatA complex formation [58,163]. Further characterization of Naa10 Ser37Pro revealed specific downstream Nt-acetylation defects in vivo as well as abnormal cell migration and proliferation capacity of affected fibroblasts [163]. In addition, *de novo* missense mutations in the *NAA10* gene have been identified in two independent cases of global developmental delay [165], and a truncated Naa10 protein is implicated in Lenz microphthalmia syndrome [166]. Finally, several NATs, in particular the NatA subunits Naa10 and Naa15, are dysregulated in various human cancers [167]. In most types of cancers investigated, NAT-overexpression mediates increased survival and proliferation of cancer cells. Because of its implication in human disease and cancer, NatA is a potential drug target and specific NAT-inhibitors are under development [168]. The role of Nt-acetylation during development is reviewed in another article in this issue [169].

The main feature of protein fatty acylation is to provide the target protein with hydrophobicity thus promoting membrane binding. Nt-palmitoylation is quite rare, but seems to be an important protein modification involved in signal transduction [106]. Indeed, the hydrophobic nature of palmitoyl has been found indispensable for the strength of Shh signaling [112]. A reduced pattering activity in mouse limb is observed for Shh in the absence of Nt-palmitoylation, while in *D. melanogaster* the Hh is found inactive [170,171]. In humans, an uncontrolled activation of Hh signaling pathway is linked to different types of cancer [172]. Compared to Nt-palmitoylation, Nt-myristoylation is more prevalent and the common denominator for many proteins modified in this way is their participation in cellular signaling pathways [173]. This involves subcellular targeting, protein-protein and protein-membrane interactions [4] and possibly protein structural stability [174].

To date, protein specific consequences of Nt-methylation have only been described for a subset of eukaryotic proteins [119–123]. Similar to NatA subunits, aberrant expression of NTMT1 has been reported in numerous cancer types. However, the functional understanding of NTMT1's role in cancer progression and prognosis is limited making it a focus of research [7]. Nt-methylation is *inter alia* involved in regulating protein function, specifically protein-DNA interactions, and several studies have suggested a role in chromatin conformation and segregation [119,121,123,126] and DNA repair [120]. Nt-methylation of RCC1 (regulator of chromosome condensation 1) was shown to promote association with chromatin. Both methylation-defective RCC1 mutants and NTMT1 knockdown results in decreased affinity for DNA, which causes mislocalization from chromatin and subsequent defects in spindle pole organization and chromosome missegregation [119,126]. RCC1 binds to chromatin through a bimodal attachment mechanism, where the methylated N-terminus associates with negatively charged DNA and the main protein body binds to histones H2A/H2B [119,124].

Due to limited knowledge on the Nt-ubiquitylome, the biological understanding of Nt-ubiquitylation remains limited. Vittal et al. proposed that Nt-ubiquitylation could be linked to protein quality control on stalled ribosomes, where it primes nascent polypeptides for subsequent polyubiquitylation [143]. On the contrary, Ube2w is predicted to harbor a nuclear localization signal and may therefore regulate nuclear proteins [175]. Ube2w has been found to be overexpressed in certain human cancers [176]. In that regard, Mittag and Marzahn have suggested that Nt-ubiquitylation could be involved in maintaining homeostasis of important regulatory proteins [177].

## 9 Discussion

The majority of eukaryotic protein N-termini is chemically modified by at least one of the many Nt-modifying enzymes. Multiple factors determine whether and how a given protein substrate will be modified including: i) the N-terminal amino acid sequence of the protein relative to the different specificities of the modifying enzymes, ii) the availability and subcellular localization of the different Nt-modifying enzymes under given conditions, and iii) the availability of the donor substrate (e.g., acetyl-CoA, SAM, etc.). Some of the Nt-modifying enzymes have overlapping substrate specificities (Table 2), so what governs the modification faith of individual proteins?

**Table 2:** Protein N-terminal modifications sorted by the N-terminal sequence

AA1	AA2	% abundance in pos. 2b)	Nt-modificationc)	N-terminus	Enzyme	Substrate
Met	Ala	23.1	Ac	Met-Ala-	NatE/F	
			iMet exc.	Met-Ala-	MetAP1/2	
			Ac	Ala-	NatA	
			Methyl	Ala-Pro-Lys-	NTMT1/2	DDB2, SET
	Arga)	4.5	Ac	Met-Arg-	NatE/F	
	Asn	3.2	Ac	Met-Asn-	NatB	
	Asp	5.7	Ac	Met-Asp-	NatB	
			iMet exc.	Met-Asp-	*ND*	Cyto. β-actin
			Ac	Asp-	Naa10	Cyto. β-actin
	Cys	0.9	iMet exc.	Met-Cys-	MetAP1/2	
			Ac	Cys-	NatA	
			Palm	Cys-	Hhat	Shh
	Gln	2.3	Ac	Met-Gln-	NatB/F	
	Glu	9.6	Ac	Met-Glu-	NatB	
			iMet exc.	Met-Glu-	*ND*	Cyto. γ-actin
			Ac	Glu-	Naa10	Cyto. γ-actin
	Gly	7.9	Ac	Met-Gly	NatF	
			iMet exc.	Met-Gly-	MetAP1/2	
			Ac	Gly-	NatA	
			Methyl	Gly-Pro-Lys-	NTMT1/2	CENP-A/B
			Myr	Gly-	NMT1/2	
			Palm	Gly-	PAT	Gα_S_
	Hisa)	1.0	Ac	Met-His-	NatE/F	
	Ile	1.5	Ac	Met-Ile-	NatC/F	
	Leu	5.3	Ac	Met-Leu-	NatC/E/F	
	Lys	4.3	Ac	Met-Lys-	NatE/F	
	Met	1.6	Ac	Met-Met-	NatE/F	
	Phe	1.8	Ac	Met-Phe-	NatC	
	Pro	5.4	iMet exc.	Met-Pro-	MetAP1/2	
			Methyl	Pro-Pro-Lys-	NTMT1/2	RB
	Ser	11.4	Ac	Met-Ser-	NatE/F	
			iMet exc.	Met-Ser	MetAP1/2	
			Ac	Ser-	NatA	
			Ac	Ser-Gly-Arg-	NatD	H2A
			Ac	Ser-Gly-Gly-	NatD	H4
			Methyl	Ser-Pro-Lys-	NTMT1/2	RCC1
	Thr	4.4	Ac	Met-Thr-	NatE/F	
			iMet exc.	Met-Thr-	MetAP1/2	
			Ac	Thr-	NatA	
	Trp	1.3	Ac	Met-Trp-	NatC	
	Tyr	0.8	Ac	Met-Tyr-	NatE/F	
	Val	4.0	Ac	Met-Val-	NatE/F	
			iMet exc.	Met-Val-	MetAP1/2	
			Ac	Val-	NatA	

a) Arg and His presumable follow the same modification pattern as Lys.

b) Distribution based on human proteins listed in Swiss-Prot 56.0.

c) Most likely, N-terminal propionylation follows the same patterns as N-terminal acetylation.

AA, amino acid; *ND*, Not determined

First, it is important to distinguish between co- and post-translational N-terminal modifications (Fig. 3). The enzymes capable of contacting the nascent N-termini, on the ribosome like MetAPs, NATs and NMTs, will be the first to target the pool of substrates. The non-ribosomal enzymes are however only capable of modifying the substrates not modified co-translationally, in addition to the neo-N-termini generated by endopeptidases. For instance, the Ser-Pro- or Gly-Pro- N-termini that are never co-translationally Nt-acetylated by NATs [60] are specifically targeted by NTMT-mediated post-translational Nt-methylation [6,123]. The substrate specificities of the NTMTs have possibly evolved to prefer those N-termini that are available (thus those that have not been modified by co-translationally acting NATs or NMTs).

This simple picture of mutually exclusive modifications may be true given that all Nt-modifications are irreversible; one modification event will normally block for further modifications, except in the case of iMet excision. However, are these N-terminal modifications truly irreversible? In most cases the Nt-modifications are probably permanent throughout the lifetime of a protein. However, it is plausible to think that at least a small fraction of the Nt-modified proteins are subjected to hitherto unidentified Nt-demodifying enzymes that act to regulate critical substrates. If so, Nt-modifications may serve a dynamic regulatory role for protein function, and different modifications may be interchangeable to serve specific functions. Substrates suggested to be subjected to Nt-demodifying enzymes include Histone H4 [178] and myosin regulatory light chain 9 (MYL9) [7,128].

Among the enzymes acting on the ribosome, there may be competition for the emerging polypeptides. Sterically, the different modifying enzymes are unlikely to bind simultaneously to the ribosome [94]. Thus a specific order of modification events could be envisioned. However, this does not always seem to be the case. For example, competition between co-translational Nt-acetylation and Nt-myristoylation has been observed and found to depend on amino acid sequences beyond the targeted Gly residue [179]. Further, a recent study suggested a kinetic competition between MetAPs and NATs: If the NAT (NatE) acts first, then the iMet will be retained since further MetAP action is precluded by the acetylated iMet, while if the MetAP acts first, then iMet excision will occur and could potentially be followed by downstream Nt-acetylation by NatA [180].

**Figure 2:**
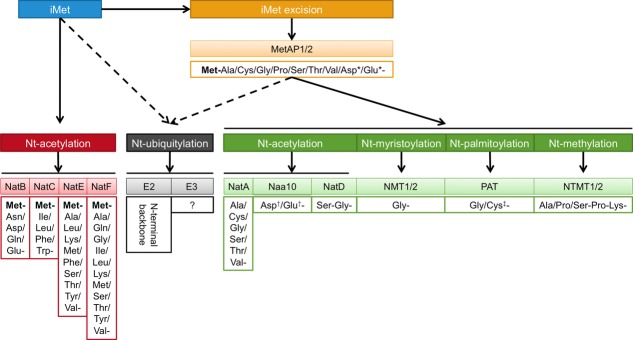
Substrate specificity of N-terminal modifying enzymes. Proteins are synthesized with an initiator methionine (iMet). The iMet can remain at the N-terminus (blue) or be removed by MetAPs (yellow). A retained iMet can undergo Nt-acetylation (red) by one of four NATs (NatB/NatC/NatE/NatF), depending upon the subsequent amino acid (listed). Following iMet removal, the N-terminal amino acid residue can become Nt-acetylated, Nt-myristoylated, Nt-palmitoylated or Nt-methylated (green). It is not known whether Nt-ubiquitylation (grey) takes place on iMet and/or the exposed amino acid residue following iMet removal. A consensus sequence for Nt-ubiquitylation has not been established. *For Met-Asp and Met-Glu of mammalian cytoplasmic β-actin and γ-actin, respectively, iMet excision is catalyzed by an unidentified aminopeptidase. †Asp and Glu of mammalian cytoplasmic β-actin and γ-actin, respectively, are Nt-acetylated by Naa10. ^ǂ^Cys is post-translationally Nt-palmitoylated after generation of protein neo-N-termini by endopeptidases.

Within a single class of enzymes like the NATs there is an apparent overlap in the in vitro substrate specificity; NatC, NatE and NatF all Nt-acetylate Met-starting N-termini with an aliphatic or hydrophilic amino acid in the second position (Fig. 2). This could perhaps be explained by specialization towards different types of substrates in vivo. NatC co-translationally acetylates hydrophobic N-termini that are normally *never* subjected to iMet excision (Met-Ile-, Met-Leu-, etc.). NatE (Naa50 bound to Naa15 and Naa10) co-translationally acetylates proteins that are *often* subjected to iMet excision (Met-Ala-, Met-Val-, etc.), but when targeted by NatE will retain its iMet in its acetylated state [180]. NatE/Naa50 may also have a post-translational role in the cytosol or nucleus towards Met-Leu- starting N-termini and similar substrates [49,59]. NatF, on the other hand, was very recently revealed to specifically Nt-acetylate transmembrane proteins [57]. This unique activity is most likely mediated in a post-translational manner via the membrane anchoring properties of NatF.

**Figure 3:**
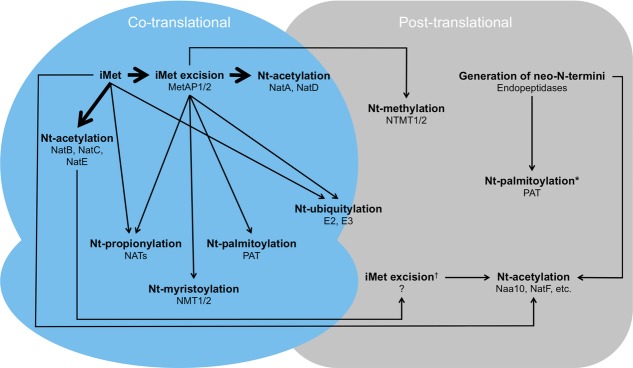
Overview of co- and post-translational N-terminal modifications. Co-translational protein modifications (left) taking place on the ribosomes include iMet excision, Nt-acetylation, Nt-propionylation, Nt-myristoylation or Nt-palmitoylation. Post-translational modifications (right) include Nt-methylation, Nt-palmitoylation or Nt-acetylation. Regarding Nt-ubiquitylation, it is uncertain whether it is a co- or post-translational event. Listed in relation to each modification are the responsible enzymes. The arrows are weighed according to the presumed extent of the individual modifications.*Preceding post-translational Nt-palmitoylation of Cys, a signal sequence is removed by endopeptidases thus generating neo-N-termini. ^†^The case of mammalian cytoplasmic β-actin and γ-actin (Met-Asp and Met-Glu, respectively) involves co-translational Nt-acetylation of iMet, presumably by NatB, followed by post-translational Ac-iMet excision by an unknown aminopeptidase and finally Nt-acetylation by Naa10.

Significant knowledge on the different N-terminal modifications and their responsible enzymes has been gained in the past decades. N-terminal modifications undoubtedly have a pivotal role in protein regulation and cellular signaling. However, future efforts are required to fully comprehend the functional role and the in vivo impact of these modifications, which includes understanding their dynamics, potential reversibility and interplay. Dysfunction or dysregulation of N-terminal modifying enzymes is implicated in human diseases including cancer, and this further stresses the importance of continuing research efforts in this field.

## Abbreviations

CoA: coenzyme A
iMet: initiator methionine
MetAP: methionine aminopeptidase
Naa: Nα-acetyltransferase
NAT: N-terminal acetyltransferase
NMT: N-terminal myristoyltransferase
NPT: N-terminal propionyltransferase
Nt: N-terminal
NTMT: N-terminal methyltransferase
PAT: palmitoylacyltransferase
SAM: S-adenosyl methionine
